# Detection of *bla*_CTX-M_ and *bla*_DHA_ genes in stool samples of healthy people: comparison of culture- and shotgun metagenomic-based approaches

**DOI:** 10.3389/fmicb.2023.1236208

**Published:** 2023-08-31

**Authors:** Edgar I. Campos-Madueno, Claudia Aldeia, Vincent Perreten, Parham Sendi, Aline I. Moser, Andrea Endimiani

**Affiliations:** ^1^Institute for Infectious Diseases (IFIK), University of Bern, Bern, Switzerland; ^2^Graduate School for Cellular and Biomedical Sciences, University of Bern, Bern, Switzerland; ^3^Institute of Veterinary Bacteriology, University of Bern, Bern, Switzerland

**Keywords:** ESBL, AmpC, stool, metagenomics, Illumina, enrichment

## Abstract

We implemented culture- and shotgun metagenomic sequencing (SMS)-based methods to assess the gut colonization with extended-spectrum cephalosporin-resistant *Enterobacterales* (ESC-R-*Ent*) in 42 volunteers. Both methods were performed using native and pre-enriched (broth supplemented with cefuroxime) stools. Native culture screening on CHROMID^®^ ESBL plates resulted in 17 positive samples, whereas the pre-enriched culture (gold-standard) identified 23 carriers. Overall, 26 ESC-R-*Ent* strains (24 *Escherichia coli*) were identified: 25 CTX-M and 3 DHA-1 producers (2 co-producing CTX-Ms). Using the SMS on native stool (“native SMS”) with thresholds ≥60% for both identity and coverage, only 7 of the 23 pre-enriched culture-positive samples resulted positive for *bla*_CTX-M_/*bla*_DHA_ genes (native SMS reads mapping to *bla*_CTX-M_/*bla*_DHAs_ identified in gold-standard: sensitivity, 59.0%; specificity 100%). Moreover, an average of 31.5 and 24.6 antimicrobial resistance genes (ARGs) were detected in the 23 pre-enriched culture-positive and the 19 negative samples, respectively. When the pre-enriched SMS was implemented, more *bla*_CTX-M_/*bla*_DHA_ genes were detected than in the native assay, including in stools that were pre-enriched culture-negative (pre-enriched SMS reads mapping to *bla*_CTX-M_/*bla*_DHAs_ identified in gold-standard: sensitivity, 78.3%; specificity 75.0%). In addition, the pre-enriched SMS identified on average 38.6 ARGs/sample, whereas for the corresponding native SMS it was 29.4 ARGs/sample. Notably, stools resulting false-negative by using the native SMS had lower concentrations of ESC-R-*Ent* (average: ~10^5^ vs. ~10^7^ CFU/g) and *E. coli* classified reads (average: 193,959 vs. 1.45 million) than those of native SMS positive samples. Finally, the detection of *bla*_CTX-M_/*bla*_DHA_ genes was compared with two well-established bioinformatic tools. In conclusion, only the pre-enriched SMS assured detection of most carriers of ESC-R-*Ent*. However, its performance was not comparable to the pre-enriched culture-based approach.

## 1. Introduction

The prevalence of people colonized at intestinal level with extended-spectrum cephalosporin-resistant *Enterobacterales* (ESC-R-*Ent*) has reached concerning levels (Bezabih et al., 2021). For instance, 21.1% of patients and 17.6% of healthy individuals worldwide carry extended-spectrum β-lactamase (ESBL)-producing *Escherichia coli* in their intestinal tract. Moreover, during the period 2001–2020, colonization rates increased 3-fold (from 7 to 25.7%) and 10-fold (from 2.6 to 26.4%) in health-care and community settings, respectively (Bezabih et al., 2022).

It is well-known that hospitalized individuals who carry at gut level ESC-R-*Ent* may contribute to the spread of these pathogens and, more importantly, can develop difficult-to-treat infections with high morbidity and mortality rates (Schwaber and Carmeli, 2007; Ling et al., 2021; Campos-Madueno et al., 2023). Conversely, data regarding the ESC-R-*Ent* intestinal colonization in healthy people is limited to prevalence surveys, mostly focusing on travelers returning from endemic regions (Bernasconi et al., 2016; Pires et al., 2016; Voor In 't Holt et al., 2020; Moser et al., 2021a,b). These individuals are also at risk of developing infections (e.g., urinary tract infections) and/or transmitting their ESC-R-*Ent* to people who are in close contact with them (e.g., in the household) (Valverde et al., 2008; Banerjee et al., 2013; Soraas et al., 2013; Tham et al., 2013; Arcilla et al., 2017). In this overall scenario, rapid and accurate recognition of ESC-R-*Ent* intestinal carriers represents an important public-health task. In fact, since current strategies to decolonize ESC-R-*Ent* carriers are not effective (Campos-Madueno et al., 2023), prompt implementation of countermeasures to prevent transmission of ESC-R-*Ent* are essential in both community and hospitals (e.g., infection prevention and control, IPC) (Tacconelli et al., 2014; Tschudin-Sutter et al., 2017).

For patients referring to the hospital setting, rapid and valid multiplex molecular screening assays for ESC-R-*Ent* may be implemented [e.g., those PCR-based, (Campos-Madueno et al., 2023)]. However, in most clinical microbiology laboratories, routine screening of the intestinal tract is still performed using culture-based methods; this is particularly true for individuals in the community (e.g., returning travelers) (Campos-Madueno et al., 2023). In these culture-based approaches, native stool or perianal/perirectal swab samples are directly plated on selective chromogenic media (Blane et al., 2016; Girlich et al., 2019). A broth pre-enrichment step (with or without antibiotics) may also be added before plating to increase the detection rate of ESC-R-*Ent* (Blane et al., 2016; Jazmati et al., 2016, 2017; Rondinaud et al., 2020; Gallah et al., 2023). Though, this approach is seldom implemented due to its increased hands-on time and turnaround time (TAT).

Recently, shotgun metagenomic sequencing (SMS) has been proposed as an alternative to culture-based methods with promising diagnostic capability (Ko et al., 2022; Purushothaman et al., 2022). However, most studies focusing on the detection of antimicrobial resistance genes (ARGs) have been conducted in the hospital setting (e.g., samples from patients with comorbidities) (Gigliucci et al., 2018; Mu et al., 2019; Leggett et al., 2020; Afridi et al., 2021; Yee et al., 2021). In contrast, few studies have directly analyzed the presence of ARGs in fecal samples of healthy people in the community by SMS (Bengtsson-Palme et al., 2015; Guernier-Cambert et al., 2021). In an Australian study, Guernier-Cambert et al. used the SMS to screen for ARGs in healthy women (*n* = 7) and infants (*n* = 2), identifying 64 unique ARGs shared across all samples. However, no culture-based methods were used to confirm the resistance phenotypes of the detected ARGs (Guernier-Cambert et al., 2021). In another analysis, Bengtsson-Palme et al. studied the resistome of 35 Swedish travelers by both culture and SMS methods finding an increased abundance of ARGs after travel. Nevertheless, ESBL encoding genes (i.e., *bla*_CTX-Ms_) were not detected by SMS, despite the fact that stool samples were culture-positive for CTX-M-producing *E. coli* strains (Bengtsson-Palme et al., 2015). In the UK, Dallman et al. used SMS to investigate the prevalence of ARGs and gut microbiota patterns in travelers (*n* = 50) at different time points (pre-travel, post-travel and follow-up). However, culture-based methods were not used to confirm the findings of the SMS analysis (Dallman et al., 2023).

In the present study, we simultaneously used culture-, PCR-, and SMS-based methods to assess the intestinal ESC-R-*Ent* colonization status of healthy individuals. In particular, we evaluated the ability of SMS as a potential tool to detect ESBL and plasmid-mediated AmpC (pAmpC) *bla* genes directly from the stool samples. Results were contextualized considering several variables such as pre-enrichment of the stool sample, gut microbiota pattern, concentrations of the colonizing ESC-R-*Ent* (if any), and detection threshold cutoffs.

## 2. Methods

### 2.1. Study design

Forty-two stool samples from volunteers were collected throughout 2021 and included in the present study. This analysis is part of an ongoing study on intestinal colonization of Swiss expats.^1^ Individuals living abroad for ≥3 months and who were ≥ 18 years old met the study inclusion criteria. At enrollment, volunteers received instructions, consent forms, shipping material and an epidemiological questionnaire. Volunteers were invited to ship their samples to the Institute for Infectious Diseases (IFIK; Bern, Switzerland) via ordinary post in collection vessels and transport containers within maximum 7 days from the return to Switzerland (e.g., to spend holidays). The study was approved by the ethical committee of the Canton Bern (ID: 2020-01683).

### 2.2. Culture-based processing of stool samples

At arrival to the IFIK, stool samples were screened for ESC-R-*Ent* by both direct and pre-enriched culture-based methods (for simplicity referred hereafter as “native culture” and “pre-enriched culture,” respectively). First, a stool aliquot (~50–100 μg) was resuspended in 1 mL saline solution (sodium chloride, 0.85%), then an undiluted 100 μL aliquot was directly plated onto a CHROMID^®^ ESBL (bioMérieux) agar plate and incubated overnight at 36 ± 1°C. This approach has an estimated limit of detection (LOD) for ESC-R-*Ent* of 1–2 10^1^ colony forming units (CFU)/100 mg of stool (Naas et al., 2011).

In parallel to the native culture, a second stool aliquot (~50–100 μg) was pre-enriched for 6 h in 10 mL Luria-Bertani (LB) broth supplemented with cefuroxime (30 μg disk, BD BBL^™^ Sensi-Disc^™^) as previously done (Bernasconi et al., 2016; Budel et al., 2019; Moser et al., 2021a,b). The pre-enrichment culture was then diluted to 0.5 McFarland, plated (100 μL) onto a CHROMID^®^ ESBL plate and incubated overnight at 36 ± 1°C. CHROMID^®^ ESBL selective plates are referred henceforward as “ESBL-plates.”

### 2.3. Species identification and antimicrobial susceptibility tests (ASTs)

ID for growing colonies (at least 1 on the ESBL-plates) was achieved using the matrix-assisted laser desorption/ionization time-of-flight mass spectrometry (MALDI-TOF MS; Bruker). ASTs were performed on broth microdilution with the Sensititre GNX2F panel (Thermo Fisher Scientific). Results were interpreted according to the 2022 EUCAST breakpoints (v12.0).^2^ *Ent* confirmed to be ESC-R (i.e., MICs >1 μg/mL for ceftriaxone, ceftazidime, cefotaxime, or cefepime) underwent whole-genome sequencing.

### 2.4. Bacterial carriage concentration

ESC-R-*Ent* carriage was determined directly from the native stool. CFU/g counts were performed in 10^−1^ serial dilutions in 1 mL saline sterile solution and plated (100 μL) onto ESBL-plates followed by overnight incubation at 36 ± 1°C. An average of 3 independent measurements was used for the final CFU/g calculation per stool sample.

### 2.5. Whole-genome sequencing for culture strains

Genomic DNA (gDNA) for WGS was extracted from the isolated ESC-R-*Ent* strains with the Invitrogen^™^ PureLink^™^ Microbiome DNA purification kit (ThermoFisher Scientific) as previously described (Campos-Madueno et al., 2021). Quality and quantity of the gDNA were assessed by Nanodrop^™^ and Qubit^™^ 3 (ThermoFisher Scientific). WGS was conducted using Illumina libraries prepared with the NEBNext^®^ Ultra^™^ II DNA library prep kit on the NovaSeq 6000 platform (2 × 150 bp paired-end output) by Eurofins Genomics (Ebersberg, Germany). Briefly, raw reads were quality checked and trimmed with FastQC v0.11.9^3^ and Trimmomatic v0.36^4^ with default parameters, respectively. Draft assemblies were generated with Unicycler v0.4.8^5^ using the trimmed Illumina paired-end reads as input.

### 2.6. Shotgun metagenomic sequencing

gDNA isolation from native stool was performed as described in the section above, but following the protocol for fecal samples from the Invitrogen^™^ PureLink^™^ Microbiome DNA purification kit using ~200 mg of starting material. For the gDNA isolation from the pre-enriched stool, an 800 μL aliquot of the cefuroxime pre-enriched LB broth was used as starting material following the above protocol, but with an initial spin down step (10 min, 14,000 × g) to pellet the sample. Quality and quantity of stool and enriched gDNA isolations were assessed as described above.

SMS libraries for gDNA obtained from native and pre-enriched stool samples (referred hereafter as “native SMS” and “pre-enriched SMS,” respectively) were prepared with the NEBNext^®^ Ultra^™^ II FS DNA Library Prep Kit and sequenced on a NovaSeq 6000 platform (2 × 150-bp paired-end output, minimum depth of raw reads, 10 million, M) by Eurofins Genomics. Downstream analyses were conducted as suggested by the general guidelines described by Quince et al. (2017). The subsequent SMS raw reads were quality checked with FastQC v0.11.9. Optical duplicates were removed with BBMap v38.18^6^ using clumpify.sh with the parameters “dedupe optical dist = 12,000” followed by read trimming with Trimmomatic v0.36 set to minimum length of 60-bp. The trimmed reads were decontaminated from human reads with Kneaddata v0.10.0^7^ using both GRCh37 (hg37dec) and GRCh38 (GRCh38_noalt_decoy_as) human genome assemblies with decoy sequences. Downstream SMS analyses were conducted using only decontaminated reads.

To simulate the minimum sequence depth required to detect *bla*_CTX-M_ or *bla*_DHA_ genes (see below), the decontaminated reads of each stool (i.e., the paired FASTQ files) were sub-sampled without replacement at 10, 20, 40, 60, and 80% using the 100, 150, 200, 250, and 300 seeds with Seqtk v1.3-r106^8^, respectively. Furthermore, the same dataset (i.e., subsampled reads) was used to generate the rarefaction curves targeting all ARGs.

A positive SMS result was defined when reads corresponding to *bla*_CTX-M_ or *bla*_DHA_ genes were mapped at ≥60% coverage and ≥ 60% identity, whereas a negative SMS result corresponded to less than <60% for both (see section below).

### 2.7. *In silico* analyses

The draft assemblies generated from the WGS of culture strains (ESC-R-*Ent*) were screened for ARGs and multi-locus sequence type (MLST) using the ResFinder v4.1 and MLST v2.0 databases from the Center for Genomic Epidemiology (CGE)^9^ using default parameters. Additionally, species identification was confirmed with the TYGS server.^10^

SMS decontaminated reads were used to screen for ARGs using the command line version of ResFinder set to paired-end reads (coverage and identity thresholds set to 60%), which uses KMA v1.3.22 (CGE) to map reads to the ResFinder database by k-mer alignment (KMA) (Clausen et al., 2018). The ResFinder KMA pipeline was chosen to analyze the metagenomic reads because of its speed (i.e., in contrast to assembly-based methods) and good performance, making it an ideal candidate for fast screening tests (i.e., lower TATs) (Clausen et al., 2018). Perfect matches corresponded to ARG hits with 100% coverage and identity, whereas partial matches (indicated as “-like”) corresponded to ARG hits thresholds ≥60% but <100% for both coverage and identity.

Read counts, mapping depth and coverage statistics were calculated automatically by the output of the KMA software. Bar plots and heatmap visualizations were generated with R v4.1.2 (2021-11-01)^11^ using “ggplot2” v3.3.6 and “pheatmap” v1.0.12 packages.

A secondary screen to support the detection of *bla*_CTX-M_ and *bla*_DHA_ genes directly from reads using the ResFinder pipeline was performed using the Comprehensive Antibiotic Resistance Database (CARD) Resistance Gene Identifier (RGI)^12^ command line version to screen metagenomic short reads (bwt) with KMA as the default read aligner (RGI v5.2.0; CARD database v3.2.7).

### 2.8. Microbiome characterization

Decontaminated reads from all native and pre-enriched SMS samples were classified at the species level using exact k-mer matches with Kraken2 v2.1.1 (NCBI taxonomic database build on: 03/28/2022)^13^ with default parameters. The Kraken2 classified reads were further processed with Bracken v2.6.1 (Bracken-build: 150-bp read length and 35 k-mer length)^14^ to estimate relative abundance at the species level (Lu et al., 2022). Individual Bracken output files were merged with the “combine_bracken_outputs.py” python script from the Bracken software. The merged Bracken data was processed with the “phyloseq” v1.38.0 R package to determine and visualize the top 10 bacterial species relative abundance in all SMS samples. The Bracken classified reads corresponding to *E. coli*, *Klebsiella pneumoniae*, and *Citrobacter freundii* were extracted with “phyloseq.”

### 2.9. PCRs of *bla*_CTX-M_ and *bla*_DHA_ genes

Screening of *bla*_CTX-M_ and *bla*_DHA_ genes was also carried out by standard PCR (FastStart^™^ Taq-DNA-Polymerase; Roche) using gDNA isolated from native and pre-enriched stool samples (referred hereafter as “native PCR” and “pre-enriched PCR,” respectively). In particular, stools that carried culture strains possessing *bla*_CTX-M_ and/or *bla*_DHA_ were screened (30 cycles) with previously reported primers (Perez-Perez and Hanson, 2002; Pitout et al., 2004). According to the intensity of the bands in the agarose gels, positive PCRs were visually defined as strong (+++), intermediate (++), and weak (+).

### 2.10. Statistical analysis

Sensitivity and specificity for the detection of *bla*_CTX-M_ and/or *bla*_DHA_ genes with native and pre-enriched SMS (positive SMS) were calculated using as gold-standard the results of the pre-enriched culture (i.e., positive or negative) together with its corresponding strain WGS characterization (i.e., presence of *bla*_CTX-M_ and/or *bla*_DHA_ genes). A false-positive result in the SMS analyses was defined as the detection of *bla*_CTX-M_ and/or *bla*_DHA_ genes that were not detected with the gold-standard.

### 2.11. Data availability statement

The draft WGS assemblies for the detected strains (*n* = 26) are available in the National Center for Biotechnology Information (NCBI) under BioProject accession PRJNA931234 (BioSamples: SAMN33052292-SAMN33052317; Genomes: JAQQZM000000000-JAQQYN000000000). The human-decontaminated SMS reads for native and pre-enriched experiments (*n* = 42 and *n* = 21, respectively) are available in the NCBI Sequence Read Archive under BioProject accession PRJNA931814 (experiment run accessions: SRR23345838- SRR23345884, SRR24519239-SRR24519254).

## 3. Results

### 3.1. Characteristics of volunteers

A total of 42 healthy volunteers (21 males and 21 females with an average age of 52.3 and 46.0 years, respectively) were included in the study. The stool samples were sent to the IFIK by the participants within an average of 2 days upon their return to Switzerland. Volunteers reported living for an average duration of 3.3 years in a foreign country located in Africa, Asia, or Europe (*n* = 11, *n* = 17, and *n* = 14, respectively). Although this is not an epidemiological study aiming to establish prevalence and risk factors for ESC-R-*Ent* gut colonization, a summary of the volunteers’ demographic and clinical characteristics is depicted in Supplementary Table S1.

### 3.2. Culture screening for ESC-R-*Ent*

As shown in Table 1, screening for ESC-R-*Ent* by native culture resulted in 17 (40.5%) positive samples, whereas 23 (54.8%) positives were identified using the pre-enriched culture. Overall, ESC-R-*Ent* showed an average concentration of 3.13 × 10^7^ (range: 1.78 × 10^2^–3.97 × 10^8^) CFU/g per stool sample. A total of 26 ESC-R-*Ent* strains (24 *E. coli*, one *C. freundii* and one *K. pneumomiae*) were isolated. All 26 ESC-R-*Ent* were fully susceptible to carbapenems, while 12 (46.2%), 7 (26.9%), and 18 (69.2%) were resistant to ciprofloxacin, gentamicin and trimethoprim-sulfamethoxazole, respectively (Supplementary Table S2).

**Table 1:** Screening of stool sample for the 42 healthy Swiss people living abroad.

Sample	Culture^a, b^		Species, ST and ARGs detected by WGS on culture strains^c^	Carriage (CFU/g)^d^	PCRs for *bla*_CTX-M_ / *bla*_DHA_^a, e^		SMS for *bla*_CTX-M_ / *bla*_DHA_^a, f^			Other *bla* genes detected by native SMS (total ARGs)^g^
	Native (*n* = 42)	Pre-enriched (*n* = 42)			Native (*n* = 23)	Pre-enriched (*n* = 23)	Native (*n* = 42)		Pre-enriched (*n* = 21)	
S1-CAM-01	Neg	Pos	*E. coli* ST1114: ***bla***_**CTX-M-15**_, *aadA5, dfrA17, mdf(A), mph(A), qnrS1, sul1*	NA	Neg	*bla*_CTX-M_^+^	Neg		*bla*_CTX-M-117-like_ *bla*_DHA-1_^l^	*bla*_TEM-1_*, bla*_OXA-347_*, bla*_cfxA3-like_ (*n* = 45)
S1-CAM-02	Pos	Pos	*E. coli* ST1136: ***bla***_**CTX-M-27**_, ***bla***_**DHA-1**_, *aph(3″)-Ib, aph(6)-Id, dfrA17, mdf(A), mph(A), qnrB4, sul1, sul2, tet(A)*	1.51 × 10^4^	*bla*_CTX-M_^+^/_DHA_^++^	*bla*_CTX-M_^+++^/_DHA_^+++^	*bla*_CTX-M-98-like_	*bla*_DHA-1-like_	*bla*_CTX-M-27_ *bla*_DHA-1_	*bla*_ACI-1_, *bla*_OXA-347_, *bla*_cfxA5_ (*n* = 26)
S1-CAM-04	Pos	Pos	*E. coli* ST69: ***bla***_**CTX-M-27**_, *aadA5, aph(3″)-Ib, aph(6)-Id, dfrA17, erm(B), mdf(A), mph(A), sul1, sul2, tet(A)*	5.13 × 10^5^	*bla*_CTX-M_^+^	*bla*_CTX-M_^++^	*bla*_CTX-M-98-like_		*bla*_CTX-M-27,_ *bla*_CTX-M-55-like,_ *bla*_DHA-1-like_	*bla*_ACI-1-like_*, bla*_OXA-347_*, bla*_cfxA-like_*, bla*_cfxA2-like_*, bla*_cfxA3-like_ (*n* = 33)
S1-IVC-02	Pos	Pos	*E. coli* ST10: ***bla***_**CTX-M-24**_, *aac(6′)-Ib-cr, aadA5, dfrA17, erm(B), mdf(A), mph(A), sul1, tet(B)*	3.84 × 10^7^	*bla*_CTX-M_^++^	*bla*_CTX-M_^+++^	*bla*_CTX-M-24_^i^		NP	*bla*_SHV-82-like_, *bla*_OXA-347_, *bla*_cfxA3_, *bla*_cfxA6-like_ (*n* = 52)
S1-KEN-03	Pos	Pos	*E. coli* ST5614: ***bla***_**CTX-M-15**_, *aadA5, aph(3″)-Ib, aph(6)-Id, dfrA17, mdf(A), mph(A), qnrS1, sul1, sul2, tet(A)*	3.45 × 10^5^	*bla*_CTX-M_^+^	*bla*_CTX-M_^+++^	Neg		*bla*_CTX-M-15_	*bla*_cfxA-like_, *bla*_cfxA6-like_ (*n* = 26)
S1-KEN-04	Pos	Pos	*E. coli* ST5614: ***bla***_**CTX-M-15**_, *aadA5, aph(3″)-Ib, aph(6)-Id, dfrA17, mdf(A), mph(A), qnrS1, sul1, sul2, tet(A)*	1.78 × 10^2^	Neg	*bla*_CTX-M_^+++^	Neg		*bla*_CTX-M-15_	*bla*_OXA-347-like_*, bla*_cfxA3-like_ (*n* = 25)
S1-KEN-05	Pos	Pos	*E. coli* ST131: ***bla***_**CTX-M-27**_, *aadA5, aph(3″)-Ib, aph(6)-Id, dfrA17, mdf(A), mph(A), sul1, sul2, tet(A)*	1.19 × 10^4^	Neg	*bla*_CTX-M_^+++^	*bla*_CTX-M-14-like_		*bla*_CTX-M-27_	*bla*_OXA-347-like_*, bla*_cfxA-like_*, bla*_cfxA3-like_ (*n* = 30)
S1-KEN-06	Pos	Pos	*E. coli* ST450: ***bla***_**CTX-M-15**_, *aph(3″)-Ib, aph(6)-Id, dfrA17, mdf(A), sul2, tet(A)*	1.01 × 10^5^	*bla*_CTX-M_^+^	*bla*_CTX-M_^+^	Neg		NP	*bla*_cfxA3_, *bla*_cfxA6-like_ (*n* = 26)
S1-KEN-07	Pos	Pos	*E. coli* ST1193: ***bla***_**CTX-M-15**_, *bla*_TEM-1,_ *aph(3″)-Ib, aph(6)-Id, dfrA17, mdf(A), mph(A), sul2, tet(B)*	4.73 × 10^7^	*bla*_CTX-M_^+++^	*bla*_CTX-M_^+++^	*bla*_CTX-M-15_		*bla*_CTX-M-15_	*bla*_TEM-1_, *bla*_cfxA3-like_ (*n* = 32)
S1-NGR-01	Neg	Neg	–	–	–	–	Neg		NP	*bla*_cfxA5-like_, *bla*_cfxA6-like_ (*n* = 29)
S1-NGR-02	Pos	Pos	*E. coli* ST2332: ***bla***_**CTX-M-15**_, *bla*_TEM-1,_ *dfrA7, mdf(A), sul1, tet(A)*	1.66 × 10^6^	*bla*_CTX-M_^+++^	*bla*_CTX-M_^+++^	*bla*_CTX-M-15_		NP	*bla*_ACT-4-like_*, bla*_TEM-1_*, bla*_OXA-1_*, bla*_ACI-1_*, bla*_cfxA3_ (*n* = 40)
S1-TAN-01	Neg	Neg	–	–	–	–	Neg		Neg	*bla*_TEM-1_*, bla*_cfxA4-like_*, bla*_cfxA6-like_ (*n* = 29)
S1-TAN-02	Neg	Neg	–	–	–	–	Neg		NP	*bla*_cepA-like_ (*n* = 26)
S1-ZIM-01	Neg	Pos	*E. coli* ST69: ***bla***_**CTX-M-15**_, *bla*_TEM-1,_ *aph(3″)-Ib, aph(6)-Id, dfrA14, dfrA17, mdf(A), sul2, tet(B)*	NA	Neg	Neg	Neg		Neg	*bla*_OXA-347-like_*, bla*_cfxA3-like_*, bla*_cepA-29-like_ (*n* = 24)
S1-CHI-01	Pos	Pos	*E. coli* ST69: ***bla***_**CTX-M-15**_, *bla*_TEM-1_, *aph(3″)-Ib, aph(6)-Id, dfrA7, qnrS1, mdf(A), sul1, sul2, tet(A)*	8.27 × 10^4^	Neg	*bla*_CTX-M_^+++^	Neg		*bla*_CTX-M-15_	*bla*_OXA-347-like_*, bla*_cfxA3_*, bla*_cfxA6-like_ (*n* = 39)
S1-CHI-03	Neg	Pos	*C. freundii* ST22: ***bla***_**CTX-M-3**_, *bla*_CMY-48_, *bla*_OXA-1_, *bla*_TEM-1_, *aac(3)-IIa, aac(6′)-Ib-cr, aadA2b, arr-3, catB3, cmlA1, dfrA19, ere(A), fosA3, sul1, tet(B), tet(D)*	NA	Neg	Neg	Neg		Neg	*bla*_ACI-1_*, bla*_cfxA-like_*, bla*_cfxA6-like_ (*n* = 21)
S1-HKG-01	Neg	Neg	–	–	–	–	Neg		NP	*bla*_cfiA6-like_, *bla*_cfiA14-like_, *bla*_cfxA-like_ (*n* = 26)
S1-INA-02	Pos	Pos	*E. coli* ST46: ***bla***_**CTX-M-15**_, *mdf(A), qnrS1*	6.07 × 10^3 h^	Neg	Neg	Neg		*bla*_CTX-M-13-like_ *bla*_DHA-1-like_	*bla*_TEM-1-like_*, bla*_cfxA3-like_ (*n* = 37)
			*E. coli* ST5909: ***bla***_**CTX-M-14**_, *dfrA14, fosA3, mdf(A), qnrS1, tet(A)*							
	Neg	Pos	*K. pneumoniae* ST1540: ***bla***_**CTX-M-15**_, *bla*_SHV-11_, *bla*_OXA-1_, *bla*_TEM-1_*, aac(3)-IIa, aac(6′)-Ib-cr, aadA2, aph(3″)-Ib, aph(6)-Id, catA2, dfrA12, fosA, mph(A), oqxA, oqxB, qnrS1, sul1, sul2*	–^¥^						
S1-IND-01	Pos	Pos	*E. coli* ST131: ***bla***_**CTX-M-15**_, *aadA5, dfrA17, erm(B), mdf(A), mph(A), sul1*	2.18 × 10^7 h^	*bla*_CTX-M_^+^	*bla*_CTX-M_^+++^	*bla*_CTX-M-33-like_		*bla*_CTX-M-15_	*bla*_OXY-1-2-like_, *bla*_cfxA3_, *bla*_cfxA6-like_ (*n* = 38)
			*E. coli* ST616: ***bla***_**CTX-M-15**_, *aadA5, dfrA17, mdf(A), qnrS1, sul1*							
S1-IND-02	Pos	Pos	*E. coli* ST648: ***bla***_**CTX-M-15**_, ***bla***_**DHA-1**_, *bla*_TEM-1_, *aac(3)-IIa, aph(3″)-Ib, aph(6)-Id, dfrA17, erm(B), mdf(A), mph(A), qnrB4, sul1, tet(B)*	1.86 × 10^2^	Neg	*bla*_CTX-M_^++^ *bla*_DHA_^+++^	Neg		*bla*_CTX-M-15_ *bla*_DHA-1_	*bla*_ACI-1_*, bla*_cfxA6-like_ (*n* = 21)
S1-ISR-01	Neg	Neg	–	–	–	–	Neg		NP	*bla*_cfxA-like_, *bla*_cfxA6-like_ (*n* = 22)
S1-ISR-02	Neg	Pos	*E. coli* ST69: ***bla***_**CTX-M-55**_, *dfrA14, erm(B), mdf(A), mph(A), tet(B)*	NA	Neg	Neg	Neg		Neg	*bla*_cfxA5_ (*n* = 23)
S1-JOR-02	Pos	Pos	*E. coli* ST38: ***bla***_**CTX-M-15**_, *bla*_TEM-1_, *aph(3″)-Ib, aph(6)-Id, mdf(A), mph(A), qnrS1, sul2*	5.98 × 10^3^	*bla*_CTX-M_^+^	*bla*_CTX-M_^+^	Neg		*bla*_CTX-M-28-like_	*bla*_ACI-1-like_*, bla*_cfxA3_*, bla*_cfxA6-like_*, bla*_cepA-like_ (*n* = 25)
S1-JOR-03	Neg	Neg	–	–	–	–	Neg		NP	*bla*_cfxA6-like_ (*n* = 17)
S1-KOR-01	Neg	Neg	–	–	–	–	Neg		NP	*bla*_TEM-30_ (*n* = 27)
S1-SRL-01	Neg	Pos	*E. coli* ST349: ***bla***_**DHA-1**_, *mdf(A), mph(A), tet(B), qnrB4, sul1*	NA	*bla*_DHA_^++^	*bla*_DHA_^+++^	Neg		*bla*_DHA-1_	*bla*_ACI-1-like_*, bla*_OXA-347-like_*, bla*_cfxA5-like_*, bla*_cfxA6-like_ (*n* = 25)
S1-THA-01	Pos	Pos	*E. coli* ST131: ***bla***_**CTX-M-14**_, *bla*_TEM-1_, *aac(3)-IId, aph(3″)-Ib, aph(6)-Id, mdf(A), sul2, tet(A)*	2.50 × 10^5^	Neg	*bla*_CTX-M_^+++^	*bla*_CTX-M-14-like_		*bla*_CTX-M-14_	*bla*_OXA-347_*, bla*_TEM-1-like_*, bla*_cfxA4_*, bla*_cfxA6-like_ (*n* = 39)
S1-THA-02	Neg	Neg	–	–	–	–	Neg		*bla*_DHA-1-like_	*bla*_OXA-212-like_*, bla*_OXA-280-like_*, bla*_cfxA4-like_ (*n* = 31)
S1-ESP-01	Neg	Neg	–	–	–	–	Neg		NP	*bla*_cfxA5-like_, *bla*_cepA-49-like_ (*n* = 34)
S1-ESP-02	Neg	Pos	*E. coli* ST1193: ***bla***_**CTX-M-15**_, *bla*_OXA-1_, *aac(3)-IIa, aac(6′)-lb-cr, aph(3″)-Ib, aph(6)-Id, dfrA17, mdf(A), sul2, tet(B)*	NA	*bla*_CTX-M_^+^	*bla*_CTX-M_^+++^	*bla*_CTX-M-28-like_		*bla*_CTX-M-15_	*bla*_cfxA5-like_ (*n* = 23)
S1-ESP-03	Pos	Pos	*E. coli* ST69: ***bla***_**CTX-M-15**_, *mdf(A), qnrS1*	3.97 × 10^8^	*bla*_CTX-M_^+++^	*bla*_CTX-M_^+++^	*bla*_CTX-M-15_		NP	*bla*_ACI-1_*, bla*_OXA-347_*, bla*_cfxA4_*, bla*_cfxA6-like_ (*n* = 32)
S1-GBR-01	Neg	Neg	–	–	–	–	Neg		NP	*bla*_OXA-347_*, bla*_cfxA-like_ (*n* = 24)
S1-GBR-03	Neg	Neg	–	–	–	–	Neg		NP	*bla*_OXA-347_*, bla*_cfxA3-like_ (*n* = 23)
S1-GER-01	Neg	Neg	–	–	–	–	Neg		NP	*bla*_ACI-1_*, bla*_cfxA3_*, bla*_cfxA6-like_ (*n* = 23)
S1-GER-02	Neg	Neg	–	–	–	–	Neg		NP	*bla*_OXA-347-like_*, bla*_cfxA3-like_*, bla*_cfxA6-like_ (*n* = 22)
S1-GER-03	Neg	Neg	–	–	–	–	Neg		NP	*bla*_cepA_ (*n* = 18)
S1-GER-04	Neg	Neg	–	–	–	–	Neg		NP	*bla*_ACI-1_*, bla*_OXA-347_*, bla*_cfxA3-like_ (*n* = 30)
S1-GER-05	Neg	Neg	–	–	–	–	Neg		NP	*bla*_OXA-347_*, bla*_cfxA-like_*, bla*_cepA-49-like_ (*n* = 27)
S1-GER-06	Neg	Neg	–	–	–	–	Neg		NP	*bla*_OXA-347_ (*n* = 24)
S1-ITA-01	Neg	Neg	–	–	–	–	Neg		NP	*bla*_cfxA-like_ (*n* = 21)
S1-NED-01	Neg	Neg	–	–	–	–	Neg		*bla*_DHA-4-like_	None (*n* = 15)
S1-SRB-02	Pos	Pos	*E. coli* ST69: ***bla***_**CTX-M-15**_, *mdf(A), qnrS1*	2.57 × 10^7^	*bla*_CTX-M_^+++^	*bla*_CTX-M_^+++^	*bla*_CTX-M-15_		NP	*bla*_ACI-1-like_*, bla*_cfxA4-like_*, bla*_cfxA6-like_ (*n* = 33)

Results of culture-, PCR- and shotgun metagenomic sequencing (SMS)-based approaches.

Pos, positive; Neg, negative; *bla*, β-lactamase gene; ST, sequence type; ARGs, antimicrobial resistance genes; WGS, whole-genome sequencing; CFU/g, colony forming units per gram of stool; ST, sequence type; NA, not assessable; −, not applicable; NP, not performed.

^a^ In colonized subjects, positive and negative results are highlighted in green and red, respectively. A positive SMS was defined when *bla*_CTX-M_ or *bla*_DHA_ genes were present at ≥60% coverage and ≥ 60 identity; matches below the thresholds (<60%) are indicated in yellow.

^b^ Both using the ESBL-plate (CHROMID^®^ ESBL, bioMérieux). Pre-enrichment for 6 h in LB broth supplemented with cefuroxime. A “positive” result corresponds to an ESC-R-*Ent* isolated from the ESBL-plate.

^c^ Only full-length ARGs are shown. *bla*_CTX-M_ and *bla*_DHA_ genes are shown in bold.

^d^ Colony counts were determined considering morphologically similar colonies (NA corresponds to no colony growth with the native culture; ^¥^, no *K. pneumoniae* colonies were morphologically distinguishable).

^e^ The same gDNA was used for native SMS. The PCR was defined as positive when a band was present: strong (^+++^), intermediate (^++^), weak (^+^); see Supplementary Figure S1 for PCR gel results.

^f^ Partial matches are indicated with “-like”.

**Figure 1:**
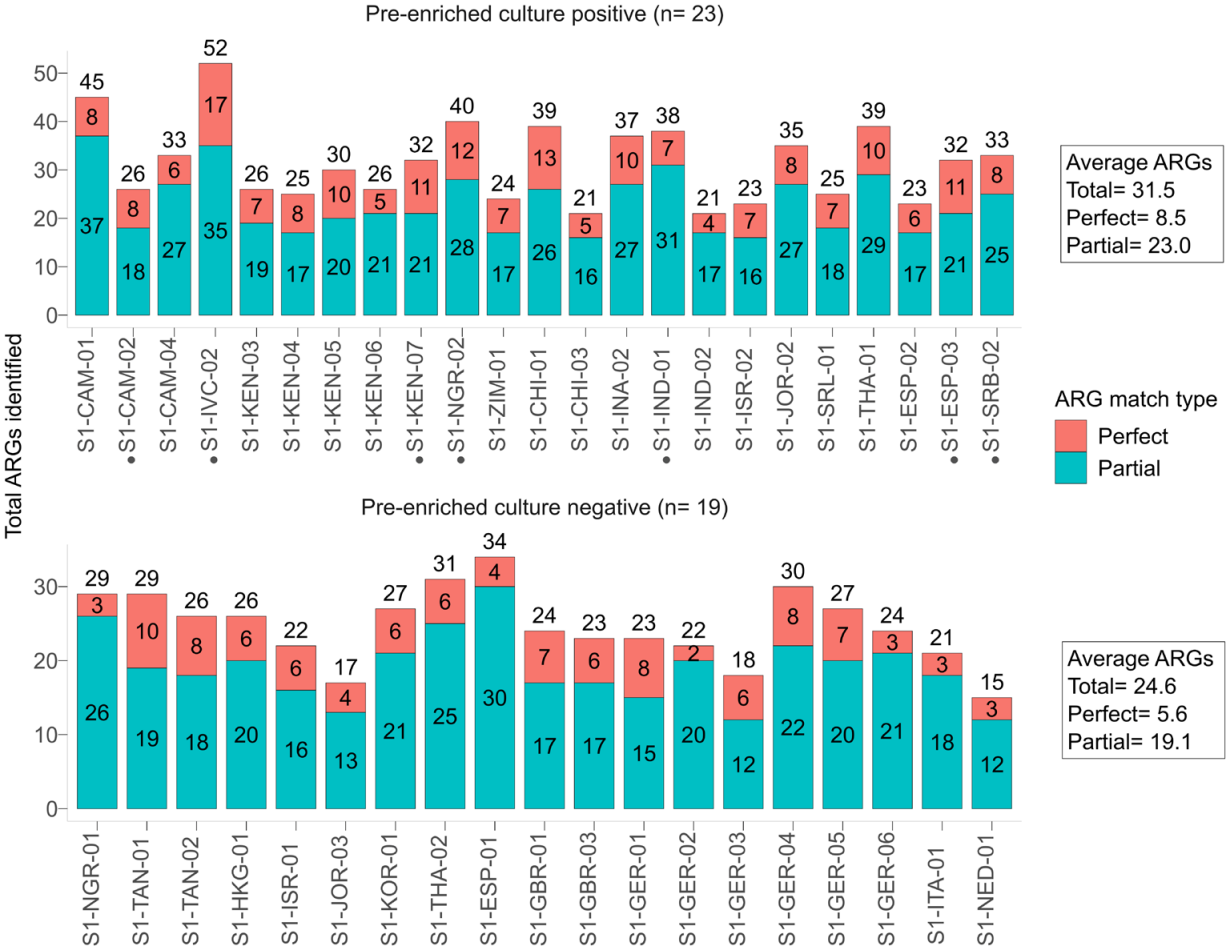
Antibiotic resistance genes (ARGs) identified by native SMS, plotted by pre-enriched culture screening result. The total number of ARGs counts and the sample are shown on the *y*-axis and *x*-axis, respectively. ARG counts are separated by match type according to the legend and shown in the bars (total count at top); a perfect match corresponds to 100% coverage and 100% nucleotide identity, while a partial match corresponds to ≥60% coverage and ≥ 60% nucleotide identity. See Supplementary File S1 for the list of all identified ARGs. Samples with a black circle (•) are those in which *bla*_CTX-M_ genes were identified by SMS at ≥60% coverage and identity threshold (*n* = 7).

^g^ For other non-*bla* ARGs detected by native SMS see Supplementary File S1. For the ratio between perfect and partial gene match see Figure 1.

^h^ Count includes both *E. coli* strains.

^i^ *bla*_CTX-M-24_ is shown according to the CARD-RGI protein database (perfect match: 100% coverage and identity; GenBank: AAN38836.1). The ResFinder result, top match being *bla*_CTX-M-14_ (GenBank: AF252622; 100% coverage and 99.89% identity), is shown in Supplementary File S1.

^l^ In this pre-enriched SMS, *bla*_DHA-1_ and *mcr-1.26*-like genes (Supplementary File S1) were also detected, although not present in the culture WGS.

### 3.3. Molecular characteristics of ESC-R-*Ent*

WGS on culture strains revealed that 6 out of 24 ESC-R *E. coli* strains were of ST69, but further pandemic clones were also detected (e.g., ST131, *n* = 3). In particular, 23 *bla*_CTX-Ms_- and 3 *bla*_DHA-1_-positives (2 co-harboring *bla*_CTX-Ms_) were found. Moreover, 2 volunteers carried CTX-M-producing *C. freundii* or *K. pneumoniae* strains (Table 1).

### 3.4. Detection of *bla* genes by native PCRs

For the 23 pre-enriched culture-positive samples, native PCRs detected only 13 (56.5%) positives for *bla*_CTX-Ms_ and/or *bla*_DHA_ (one co-harboring *bla*_CTX-M_) (Table 1, highlighted in green). The band intensities of the PCR products for 12 *bla*_CTX-M_-positives were strong/intermediate (*n* = 5) and weak (*n* = 7), while intermediate bands were present for 2 *bla*_DHA_-positives (Supplementary Figure S1).

### 3.5. Detection of *bla* genes by pre-enriched PCRs

The 23 pre-enriched culture-positive samples were also tested by pre-enriched PCRs. As a result, 6 out of 10 samples that resulted negative for *bla*_CTX-M_/*bla*_DHA_ genes by using the native PCR were actually positive using the pre-enriched approach (Table 1). As shown in Supplementary Figure S1, the band intensities for the PCR products were mostly strong/intermediate (5 out of 6).

### 3.6. Detection of ARGs by native SMS

The native SMS for the 42 stool samples resulted in an average of 13.8 M reads [phred quality score (Q) range for forward and reverse reads: Q26-Q37 (~99% of reads ≥Q30)] that were used for downstream analyses (Supplementary Table S3; Supplementary Table S4). On average, each of the 23 pre-enriched culture-positive samples contained 31.5 ARGs, whereas those of the 19 that were pre-enriched culture-negative possessed 24.6 ARGs (Figure 1; Supplementary Figure S2; Supplementary File S1).

**Figure 2:**
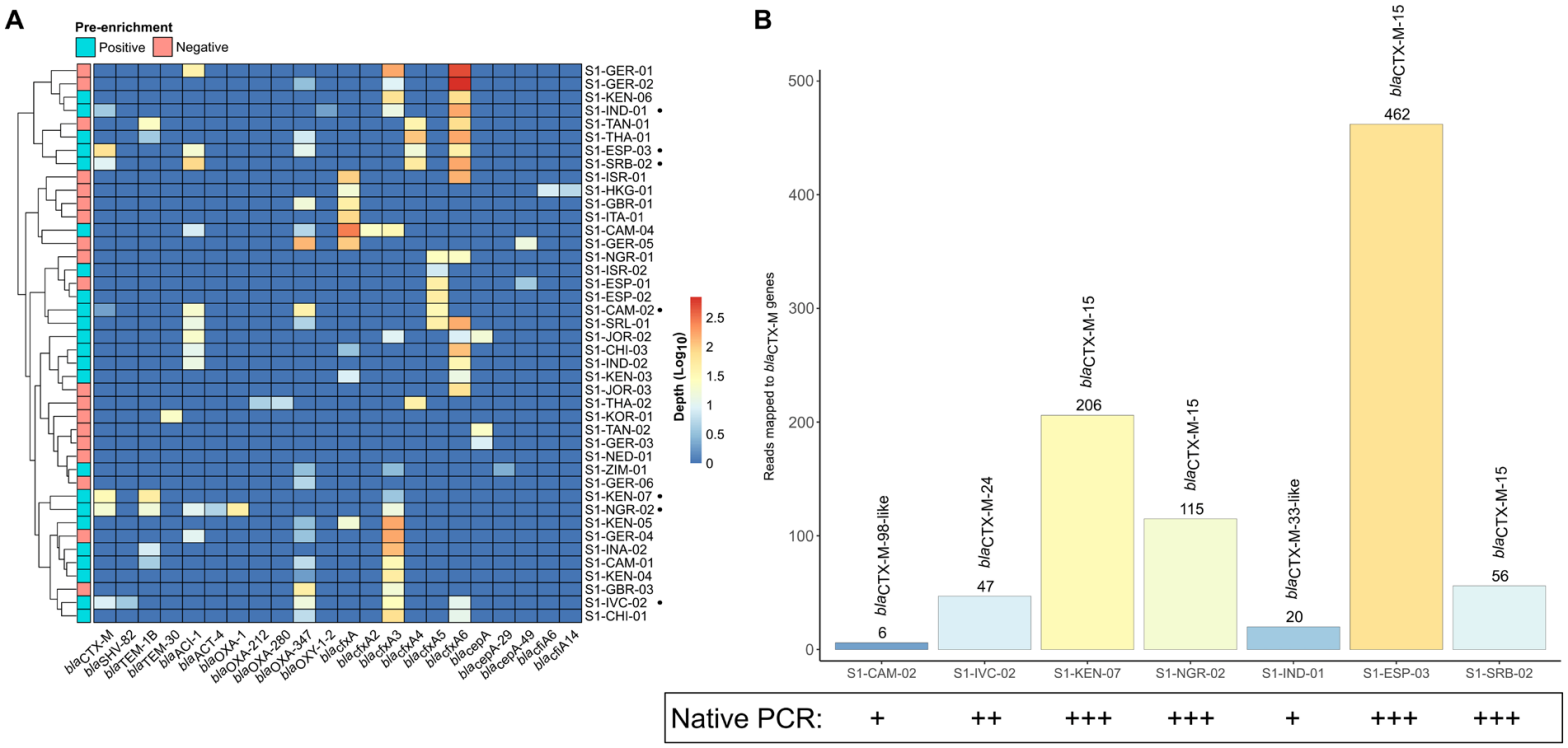
β-lactamase (*bla*) coding genes identified by native shotgun metagenomic sequencing (SMS). **(A)** Cluster matrix showing the mapping depth of the *bla* genes identified per sample. Pre-enrichment culture screening results are noted on the legend. Sample names and *bla* genes are shown on the *y*- and *x*-axis, respectively. Sample names marked by a black circle (•) correspond to *bla*_CTX-M_-positive samples by native SMS (≥60% identity and coverage threshold; *n* = 7). **(B)** Total number of mapped reads to the *bla*_CTX-M_ genes identified by native SMS. The total number of mapped reads to a *bla*_CTX-M_ gene per sample is shown above the bar and are colored by mapping depth (See Supplementary File S1 for depth, nucleotide identify and coverage). Corresponding native PCR results (intensity of bands) is also shown.

Only 7 samples that were pre-enriched culture *bla*_CTX-M_-positives were also detected by the native SMS (Table 1, highlighted in green; Figure 2A). In particular, 5 of these 7 SMS positive samples showed perfect matches (SI-IVC-02, S1-KEN-07, S1-NGR-02, S1-ESP-03, and S1-SRB-02 with *bla*_CTX-M-15/24_ genes). Two other samples with partial *bla*_CTX-M_ matches were S1-CAM-02 (*bla*_CTX-M-98-like_) and S1-IND-01 (*bla*_CTX-M-33-like_) (Table 1; Supplementary File S1). Overall, sensitivity and specificity for the native SMS were 59.0 and 100%, respectively.

Notably, at below the defined threshold (≤60% identity and coverage), 5 native SMS negative samples turned out to be positive for *bla*_CTX-Ms_ or *bla*_DHAs_, but with 24–46% identity and coverage (Table 1, highlighted in yellow).

Finally, to confirm our results obtained using the ResFinder platform, a supplementary screening with the CARD database (RGI using protein homology models) also detected the same *bla*_CTX-M_ and/or *bla*_DHA_ genes with good agreement, except for two samples (i.e., S1-IND-01 and S1-SRB-02) where the *bla*_CTX-Ms_ were not identified (Supplementary File S1).

### 3.7. Native PCR and native SMS: impact of ESC-R-*Ent* concentration in stool

Using the same gDNA, PCR yielded *bla*_CTX-M_ amplicons with band intensities proportional to the presence (read mapping) of *bla*_CTX-Ms_ in native SMS (Figure 2B).

In the 5 out of the 7 native SMS *bla*_CTX-M_-positive samples, strong/intermediate bands corresponded to perfect *bla*_CTX-M-15/−24_ matches in the SMS analysis. In these 5 samples, the number of SMS mapped reads for *bla*_CTX-Ms_ were at least 47 and the ESC-R-*Ent* concentrations were 1.66 × 10^6^ CFU/g to 3.97 × 10^8^ CFU/g. In contrast, weak native PCR bands were observed in the 2 native SMS *bla*_CTX-M_-positive samples with partial *bla*_CTX-M_ coverage. Moreover, the number of SMS mapped reads for these 2 samples were ≤ 20 and the ESC-R-*Ent* stool concentration were 1.51 × 10^4^ CFU/g and 2.18 × 10^7^ CFU/g, respectively (Figure 2B; Table 1).

In the 5 native SMS samples that were negative, but contained partial *bla*_CTX-M_ or *bla*_DHA_ matches, native PCRs were intermediate/weak positive for 3 and negative for 2 of them. The ESC-R-*Ent* stool concentration of these 2 samples were 1.19 × 10^4^ CFU/g and 5.13 × 10^5^ CFU/g, respectively (Table 1).

Overall, in the native SMS, *bla*_CTX-Ms_ were detected at an average minimum depth of 976,719 reads in 3 samples (S1-KEN-07, S1-NGR-02 and S1-ESP-03) corresponding to strong PCR bands, whereas in the other 4 native SMS samples, the average minimum depth was ~5.5 M reads corresponding to intermediate/weak bands (Supplementary Figure S1; Supplementary File S1).

In the rarefaction plot, we show that the total number of ARGs per native SMS sample reached saturation at approximately ≥80–100% of the total library size (Supplementary Figure S4A; Supplementary File S1). The minimum sequencing depth (% of total library size) required to detect the *bla*_CTX-M_ genes was ≤20% for the 5 samples with strong/intermediate native PCR bands, whereas the 2 samples with weak bands needed ≥40% (Supplementary Figure S4B).

### 3.8. Detection of ARGs by pre-enriched SMS

**Figure 3:**
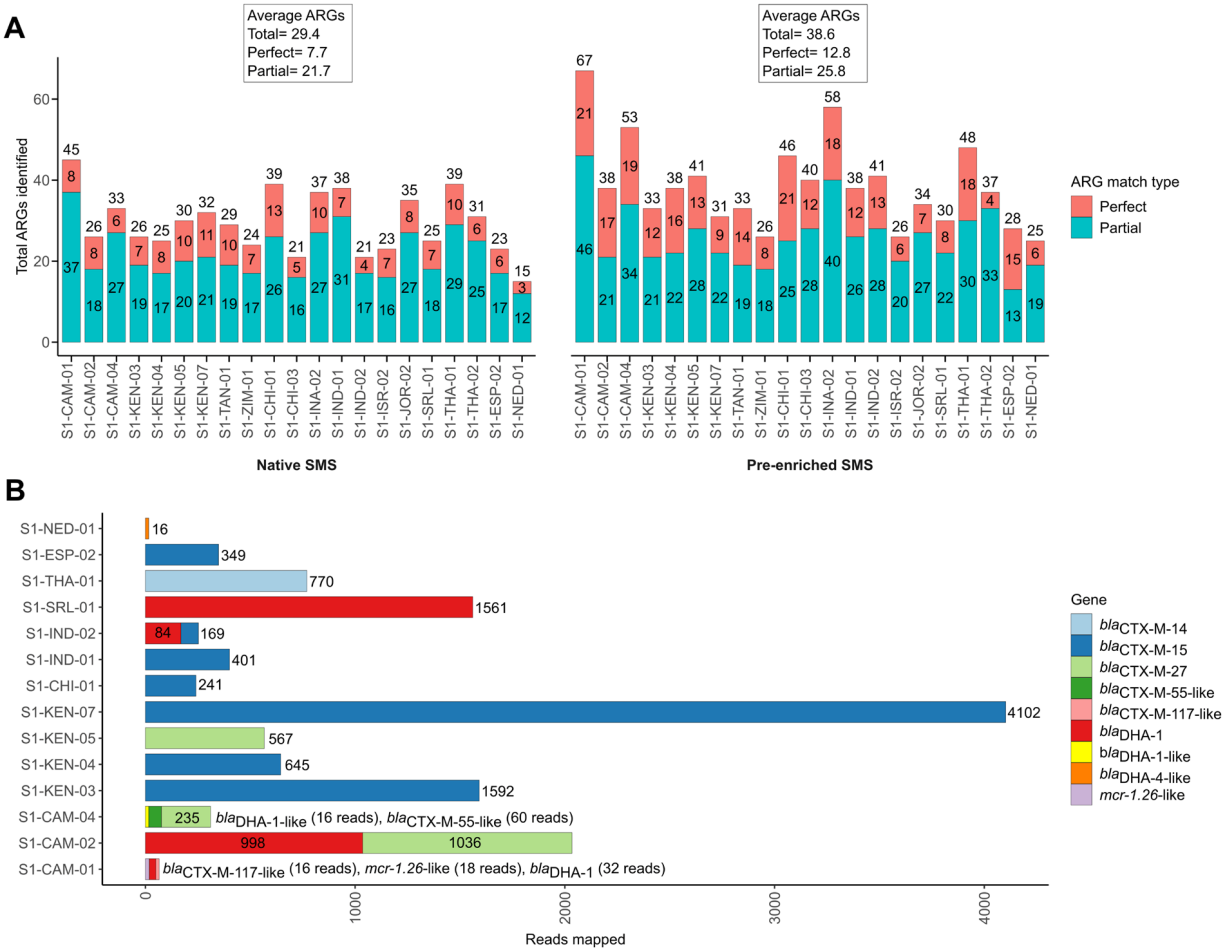
Antibiotic resistance genes (ARGs) identified by pre-enriched shotgun metagenomic sequencing (SMS) in 21 representative samples. **(A)** Comparison of ARGs identified by native and pre-enriched SMS. The total number of ARGs counts and the sample are shown on the *y*-axis and *x*-axis for both native and pre-enriched SMS, respectively. ARG counts are separated by match type according to the legend and shown in the bars (total count at top); a perfect match corresponds to 100% coverage and 100% nucleotide identity, while a partial match corresponds to ≥60% coverage and ≥ 60% nucleotide identity. See Supplementary File S1 for the list of all identified ARGs. **(B)** The *bla*_CTX-M_-, *bla*_DHA-1_- and *mcr-1*-like genes identified by using pre-enriched SMS. The total number of mapped reads per sample is shown in the bars and colored by gene (for a complete list of identified ARGs and coverage, see Supplementary File S1). Note, *bla*_DHA-4-like_ was identified in S1-NED-01 (64.04 and 64.21% identity and coverage, respectively), while below threshold (≤60% identity and coverage) reads mapping to *bla*_CTX-M-13-like_ (2 reads; 34.47% identity/coverage) and *bla*_DHA-1-like_ (2 reads; 20.26% identity/coverage), *bla*_CTX-M-28-like_ (2 reads; 34.47% identity/coverage), and *bla*_DHA-1-like_ (9 reads; 38.42% identity/coverage) were detected in S1-INA-02, SI-JOR-02, and S1-THA-02, respectively (data not shown).

To investigate whether the sensitivity of SMS to detect *bla*_CTX-M/DHA_ genes could be improved using enriched stool gDNA, we conducted SMS on 21 of the 42 samples. For the 21 samples, the pre-enriched SMS resulted in an average of ~13.1 M high quality reads (~99% of reads ≥Q30) (Supplementary Table S3; Supplementary Table S4; Supplementary Figure S3). On average, this approach identified 38.6 ARGs per sample, whereas the corresponding native SMS samples had an average of 29.4 ARGs (Figure 3A; Supplementary File S1). Notably, 18 of the 21 samples were pre-enriched culture-positive for *bla*_CTX-M_ and/or *bla*_DHA_-possessing strains but, as mentioned above, only part of them (i.e., 3/18) were correctly identified as positive with the native SMS analysis (Table 1).

With the pre-enriched SMS approach, 13 out of the 18 samples turned out to be positive for *bla*_CTX-M_/*bla*_DHA_ genes (Table 1, highlighted in green). Interestingly, 1 out of the remaining 3 samples that were pre-enriched culture-negative (S1-NED-01) resulted false-positive for a *bla*_DHA-4-like_ gene. Overall, sensitivity and specificity for the pre-enriched SMS were 78.3 and 75.0%, respectively.

Of note, two *bla*_DHA_ genes were not identified by culture in S1-CAM-01 and S1-CAM-04; the first also contained an *mcr*-1 (*mcr*-1.26-like) gene at 86.6% identity and coverage, respectively (Table 1; Figure 3B). Furthermore, two samples that resulted negative by pre-enriched SMS (S1-INA-02 and S1-JOR-02) were actually harboring *bla*_CTX-M/_*bla*_DHA_ genes, but with only 20.3–34.4% identity and coverage (Table 1, highlighted in yellow; Figure 3).

Overall, *bla*_CTX-M_ and/or *bla*_DHA_ genes were detected at an average minimum depth of 1.2 M reads in the pre-enriched SMS. In one sample (S1-CAM-01) corresponding to weak PCR bands, *bla*_CTX-M_ and *bla*_DHA_ genes were detected at an average minimum depth of 2.21 M and 4.42 M reads, respectively (Supplementary Figure S1; Supplementary File S1).

Similarly to the native SMS, the rarefaction plot shows that the total number of ARGs per pre-enriched SMS sample reached saturation at approximately ≥80–100% of the total library size (Supplementary Figure S4A). For the 13 positive samples, the minimum sequencing depth (% of total library size) required to detect *bla*_CTX-Ms_ was ≤20%, whereas that to detect *bla*_DHA_ genes ranged between 10 and 80% (Supplementary Figure S4B; Supplementary File S1).

### 3.9. Abundance of *Escherichia coli* reads in both native and pre-enriched SMS

**Figure 4:**
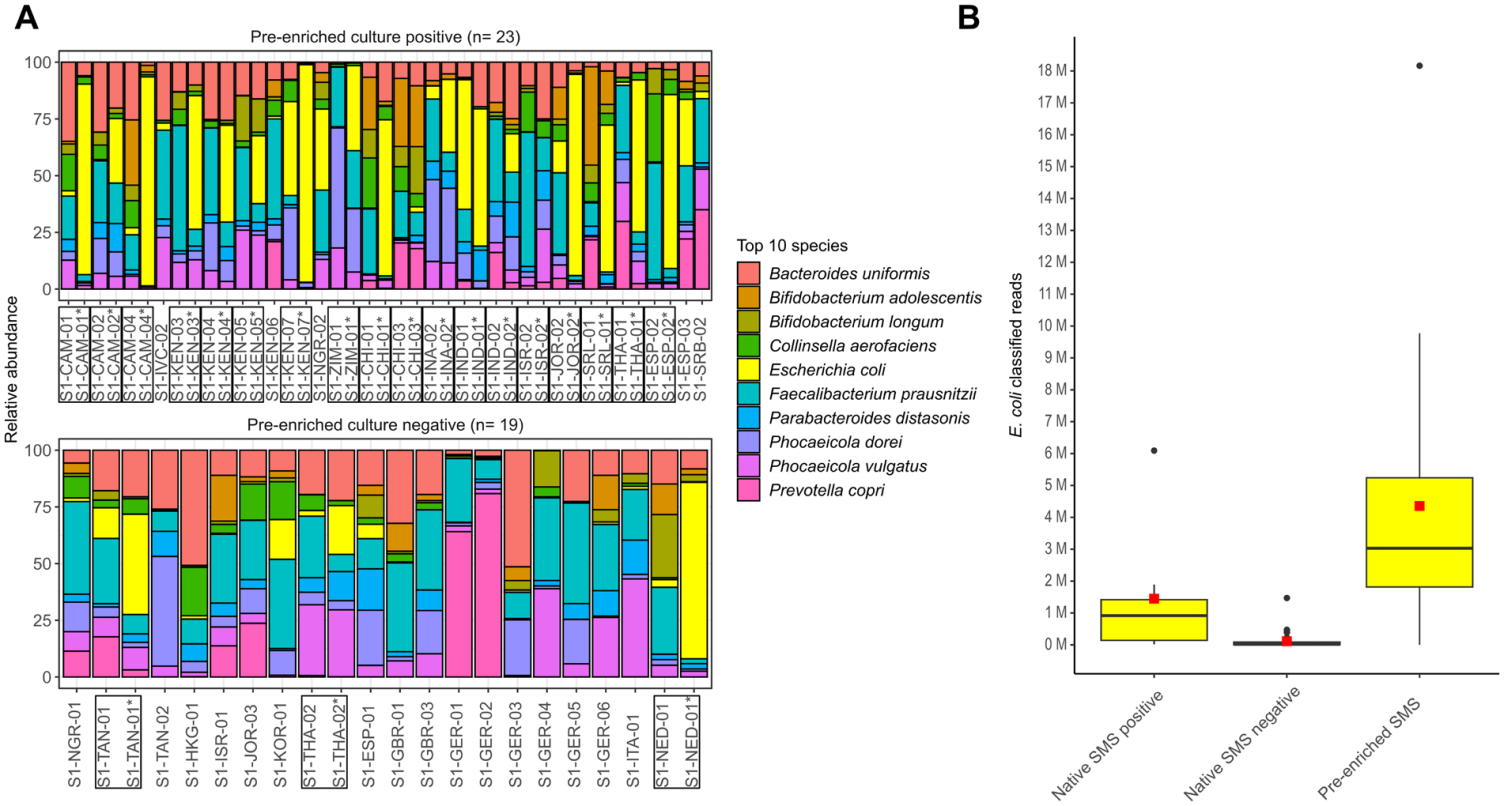
Microbiome characterization at the species level by shotgun metagenomic sequencing (SMS). **(A)** Native and pre-enriched SMS relative abundance for the top 10 bacterial species in the 23 and 19 pre-enrichment culture positive and negative stools, respectively. Results of the pre-enriched SMS are marked with an asterisk (*); both pre-enriched and matching native SMS samples are marked with a box. **(B)** Distribution of *E. coli* classified reads in the 42 native (positive: *n* = 7; negative: *n* = 35) and 21 pre-enriched SMS (positive: *n* = 14; negative: *n* = 7). The red square and black line in the box plot correspond to the average and median number of classified *E. coli* reads, respectively: 1,449,006.7 and 916,144 (native SMS positive), 113,465.7 and 20,449 (native SMS negative), 4,358,135.9 and 3,030,602 (pre-enriched SMS). See Supplementary File S1 for raw data.

In the decontaminated reads from both native and pre-enriched SMS, the top 10 bacterial species included *E. coli* (Figure 4A).

In native SMS samples that where *bla*_CTX-M_-positive (*n* = 7), the number of *E. coli* classified reads was on average of ~1.45 M, while in the 35 samples resulting native SMS negatives it was 113,466. In 10 of the 35 native SMS negatives that were both native and pre-enriched culture-positives, an average of 193,959 classified *E. coli* reads were detected. Furthermore, in the 21 pre-enriched SMS samples, the number of *E. coli* classified reads was on average of ~4.36 M (Figure 4B; Supplementary File S1).

## 4. Discussion

The SMS-based approach has been suggested as a tool for the non-targeted identification of genes from clinical samples (Chiu and Miller, 2019). However, data regarding the implementation of this technique on complex samples like the stool of people living in the community is scarce.

In this work, we evaluated the potential of SMS as a screening tool for intestinal colonization with ESC-R-*Ent* in healthy individuals and compared it with the standard culture-based methods. Specifically, we focused on the detection of *bla*_CTX-Ms_ and *bla*_DHAs_ and explored potential factors that may influence their detection with SMS. Other common acquired ESBL and AmpC *bla* genes in *Enterobacterales* (e.g., *bla*_TEM_, *bla*_SHV_ and *bla*_CMY_) were not considered due to their absence or rarity in the genome of our 26 isolated ESC-R-*Ent* (Bush and Bradford, 2020).

### 4.1. Pre-enriched culture-based method

Identification of intestinal carriers of ESBL or AmpC producers is usually achieved by native culture screened on selective chromogenic media. However, considering that the concentration of some ESC-R-*Ent* in the gut may be lower than the technical LOD (~10^1^ CFU/100 mg) and the commensal bacteria (e.g., *Bacteroides* spp.), the native culture approach may not provide sufficient resolution (e.g., resulting in visible bacterial growth in culture plates) to detect these pathogens, unless a pre-enrichment step is performed (Naas et al., 2011; Girlich et al., 2014; Rondinaud et al., 2020; Sadek et al., 2020; Gallah et al., 2023).

Our analysis confirmed that even a short selective pre-enrichment (6 h) increases the capacity to identify the ESC-R-*Ent* colonized individuals of about 15% when compared to the direct screening alone (Table 1). For this reason, the pre-enriched culture approach was used as gold-standard to evaluate the performance of the SMS method in detecting *bla*_CTX-M_ and/or *bla*_DHA_ genes.

### 4.2. Native SMS is not sufficiently sensitive to detect *bla*_CTX-M_ and *bla*_DHA_ genes

Recently, SMS has been used to identify bacterial species and ARGs such as *Clostridioides difficile*, KPC-producing *K. pneumoniae,* Shiga toxin- and CTX-M-producing *E. coli* in stool samples from hospitalized patients (Andersen et al., 2016; Schneeberger et al., 2016; Ward et al., 2016; Zhou et al., 2016; Grall et al., 2017; Gigliucci et al., 2018; Mu et al., 2019; Leggett et al., 2020; Yee et al., 2021). In contrast, only Guernier-Cambert et al. (2021), Bengtsson-Palme et al. (2015), and Dallman et al. (2023) analyzed the stool of healthy people (*n* = 9, *n* = 35, and *n* = 50, respectively) by using SMS.

As we have done in the present work, most of the herein cited studies have used the Illumina short-read native SMS, which is a well-established method. Nevertheless, despite the ability of short-read SMS to detect genetic targets, the method has a long TAT (e.g., up to 40 h sequencing run). This time lag is inappropriate for implementing IPC measures in clinical practice (Liu et al., 2021). Cost is also an issue, as most clinical laboratories do not have the infrastructure, equipment, or funding to implement short-read SMS. In addition, there are no standardized bioinformatics pipelines for pre-processing, analyzing and interpreting SMS data, making it difficult to implement in the clinical laboratory (Chiu and Miller, 2019). Therefore, because differences in results may be due only to differences in the technical approaches, comparing the performance (i.e., sensitivity) between SMS studies may not be appropriate.

In our study, comparison of the native SMS and the pre-enriched culture approaches showed that the native SMS has a very low sensitivity (59.0%) for the detection of *bla*_CTX-M/DHA_ genes in stool (Table 1, Figure 2). Other *bla* genes, such as the *bla*_cfxA-type_ from *Bacteroides* spp. were identified (Eitel et al., 2013). The presence of such *bla* genes is common due to their high abundance in the gut flora (Gatica et al., 2019). In fact, 3 species belonging to the *Bacteroidaceae* family were among the top 10 species in all native SMS samples tested in the present study (Figure 4A). Overall, considering the amount of ARGs detected (Figure 1; Supplementary File S1), SMS can be used as a non-targeted method to describe the overall resistome (Bengtsson-Palme et al., 2015). Nevertheless, in view of the arguments outlined above, gDNA from less represented species (e.g., the ESBL producers) might not be abundant enough to be detected by native SMS.

### 4.3. Impact of sequencing depth on the sensitivity of SMS

Despite the suggested low sensitivity of native SMS, it is important to note that several factors such as the sequencing depth could influence the detection of important ARGs (Santiago-Rodriguez et al., 2020). However, although various sequencing depths have been proposed (e.g., 5–50 M, including 2.5 billion ultradeep sequencing), it is not yet clear what is the optimal value required to detect targeted ARGs in stool (Hillmann et al., 2018; Zaheer et al., 2018; Liu et al., 2022).

In this study, native SMS resulted in variable sequencing depths, as well as the identification of *bla*_CTX-M_ genes which was possible at an average minimum depth between 996,719 to 5.5 M reads (Supplementary Figure S2; Supplementary Table S3; Supplementary File S1). Furthermore, *bla*_CTX-M_ genes were identified earlier at lower sequencing depths than other ARGs, confirming a high concentration of bacteria carrying such *bla* genes (Table 1; Supplementary Figure S4). Nevertheless, such differences were expected and not found to be a major factor affecting SMS sensitivity in this study. In fact, the average of reads for the native SMS corresponding to the pre-enriched culture-positive and culture-negative samples were in the same range (~13–15 M). This observation leads to the hypothesis that other factors such as the bacterial load (CFU/g) in native stool might play a more important role in the detection of ARGs, as their presence (e.g., copy number) might be reflected by the ESC-R-*Ent* carrying them.

### 4.4. Bacterial concentration may influence the outcome of native SMS

*E. coli* is estimated to be present in a wide range (3 × 10^2^ to 10^9^ CFU/g) in healthy individuals (Wotzka et al., 2018). Intestinal carriage of 3.6 × 10^7^ CFU/g of ESC-R *E. coli* (average) and up to 1 × 10^8^ CFU/g (e.g., CTX-M-15-producing *E. coli*), for example, have been reported in travelers to Vietnam and in a university hospital in Morocco, respectively (Girlich et al., 2014; Nakayama et al., 2020). Moreover, in the study by Grall et al., the *bla*_CTX-M_ genes detected by native SMS were from stool samples of adults under imipenem treatment with a median carriage load of log 9.4 CFU/g (equivalent to 2.51 × 10^9^ CFU/g) (Grall et al., 2017).

In line with the above surveys, the ESC-R-*Ent* stool concentration in the native SMS positive and negative samples in our study varied between 10^4^ to 10^8^ and 10^2^ to 10^5^ CFU/g, respectively (Table 1). Consistently, the number of *E. coli* classified reads was also variable between native SMS positive and negative samples (on average 1.45 M vs. 113,466, respectively) (Figure 4B; Supplementary File S1).

The proportion of the target bacterial pathogens to other species in the stool needs to be large enough to be captured during the gDNA isolation and following SMS processing (Thomas et al., 2012). We found that 5 positive native SMS samples mapped in full (100% coverage, range, 47–462 reads) to *bla*_CTX-M_ genes (Table 1; Figure 2B). Importantly, a simple native PCR result was indicative of a following positive native SMS result: strong bands corresponded to complete coverage of the *bla*_CTX-M_ genes (Supplementary Figure S1). Moreover, in most samples, the ESC-R-*Ent* concentration in stool was proportional to the intensity of the PCR results and to the number of mapped reads (Table 1; Figure 2B). Therefore, considering that the abundance of ESC-R-*Ent* in gDNA is variable, the predictability of a successful native SMS screening to identify important ARGs (e.g., *bla*_CTX-Ms_) may be evaluated by PCR-based tests commonly used in the diagnostic laboratory (Endimiani et al., 2020).

### 4.5. Effect of sensitivity cutoffs when using the native SMS

Another important factor to consider is the sensitivity of the parameters used (e.g., coverage cutoffs) for the *in silico* screening as they might influence the results (e.g., low abundance reads may be discarded during metagenome assembly) (Thomas et al., 2012).

We detected *bla*_CTX-M_ at 100% (*n* = 5) and at 77–90% (*n* = 2) coverage (Figure 2B; Supplementary File S1). On this note, at lower cutoffs (≤60%), 4 samples that were native SMS negative actually revealed reads mapping to *bla*_CTX-Ms_. Therefore, because of the low abundance of important ARGs, it is essential to consider that stricter cutoffs might lead to false-negative results. In this case, using the ResFinder default parameters (i.e., ≥90% identity and ≥ 60% coverage) we would have missed 2 out of 7 *bla*_CTX-M_-positives (S1-CAM-02 and S1-IND-01) in the native SMS (Table 1; Supplementary Table S1).

Remarkably, the use of different screening tools such as CARD-RGI, which uses a protein homolog-based database as opposed to the nucleotide database used by ResFinder, may affect the detection sensitivity for the *bla*_CTX-M/DHA_ genes. In fact, we found that CARD-RGI failed to detect 2 *bla*_CTX-M_-positives (S1-IND-01 and S1-SRB-02; Supplementary File S1) at ≥60% coverage as our ResFinder pipeline.

### 4.6. Pre-enriching is a potential strategy to increase the sensitivity of SMS

An alternative and cheap strategy to increase the sensitivity of SMS is to consider SMS from pre-enriched stool samples. Few studies have explored this approach with the aim of enhancing the detection of specific pathogens and ARGs present at low concentrations in stool. For example, in the study by Raymond et al., the enriched stool (7 days in both aerobic and anaerobic broths with and without cefoxitin) of 18 participants treated with cefprozil for 78 days were analyzed by SMS. They found that the long stool enrichments were necessary for the detection of less abundant bacteria and an increase in overall ARGs compared to native SMS (Raymond et al., 2019). In another recent study, a shorter stool enrichment (6 h) in combination with 3 antibiotics (vancomycin, metronidazole, and cefpodoxime) was necessary to detect carbapenemase-producing *Ent* (*bla*_NDM-1_- and *bla*_KPC-2_-positive) in spiked stools from 3 inpatients under ceftazidime exposure and one healthy volunteer (Peto et al., 2019).

Results of these exploratory studies clearly indicated that the choice of the antibiotic(s) used for the enrichment select the group of pathogens and ARGs that the SMS can detect during the analysis (i.e., narrowing down the number of hypothetical perceivable targets present in the stool). Therefore, a preliminary strategic decision based on what to look for in the sample of interest should be done prior to SMS analysis.

In our case, a pre-enrichment with cefuroxime for 6 h was specifically implemented to target the ESC-R-*Ent*. As a result, this approach was sufficient to increase the sensitivity of SMS of about 20%. In particular, the pre-enriched SMS led to the identification of *bla*_CTX-Ms_ in many samples that were not identified by native SMS (an increase of about 50–55%). An additional side benefit of the pre-enriched SMS approach was the overall lower requirement of sequencing depth (~1.2 M reads for both *bla*_CTX-M_ and *bla*_DHA_ genes) to detect *bla*_CTX-Ms/DHAs_ vs. 996,719 to 5.5 M reads in the native SMS, potentially speeding up sequencing times (Koh et al., 2023). Both *bla*_CTX-M_ and *bla*_DHA_ genes were also detected earlier at lower sequencing depths than other ARGs, supporting the observation of a high concentration of bacteria carrying such *bla* genes (Table 1; Supplementary Figure S4).

Furthermore, pre-enrichment also resulted in the identification of other important ARGs not observed in the genome of isolated ESC-R-*Ent* such as the *bla*_DHA_ genes (Table 1; Figure 3). In this regard, it can be speculated that such *bla*_DHA_ genes (especially those with thresholds ≤60%) were mostly carried by natural hosts (e.g., *Morganella morganii*) that overgrowth under the selective pressure of cefuroxime affecting the final specificity of the pre-enriched SMS analysis. This highlights the importance of the concurrent use of culture-based methods as a confirmatory test, as findings detected by SMS may not be clinically relevant (i.e., false-positives).

We finally emphasize that the stool pre-enrichment could be beneficial to improve the detection of clinically important ARGs with other DNA-based methods as well. For instance, we previously demonstrated that testing pre-enriched stools (LB broth supplemented with colistin) increased the detection rate of *mcr-1* compared to native stools by real-time PCR (Dona et al., 2017).

### 4.7. Conclusion

Our results indicate that the native SMS analysis is unable to reveal the presence of the clinically important *bla*_CTX-M_/*bla*_DHA_ ARGs in the stool of colonized individuals who have a low concentration of ESC-R-*Ent* (e.g., in healthy people in the community). With the pre-enriched SMS, a short selective incubation time and the use of detection cutoffs lower than those set by default in available analytical platforms may significantly increase the sensitivity of SMS. However, the native culture method still offers the same results, whereas the pre-enriched culture-based approach is performing significantly better in detecting ESC-R-*Ent* producing CTX-Ms and/or DHAs β-lactamases.

Although pre-enriched SMS is a promising alternative to non-targeted native SMS, further studies should investigate the effect of deep sequencing (beyond >10 M reads) with and without non-targeted pre-enriched SMS (i.e., no addition of antibiotic) and native SMS alone, since performance [i.e., sensitivity to detect specific ARGs (*bla*_CTX-M_ or *bla*_DHA_) or all present ARGs] may simply increase due to the availability of more reads or *vice versa*, as may have been the case in this study. Furthermore, the pre-analytical effects (e.g., stool collection methods, gDNA isolation kit) should be considered as they may impact the sensitivity of SMS analyses (Thomas et al., 2012; Quince et al., 2017; Guan et al., 2021).

We also emphasize that, at the moment, the routine implementation of the Illumina short-read SMS platform presents logistical challenges, high costs, and long TATs. In this context, the Nanopore long-read sequencing may represent a cheaper, faster (<6 h sequencing run) and easy to implement alternative to Illumina. Moreover, the Nanopore metagenomic output could further link the identified ARG with the corresponding bacterial species and mobile genetic element (e.g., plasmid) (Mu et al., 2019; Leggett et al., 2020). However, as opposed to Illumina technology, Nanopore requires high DNA inputs for library preparations (e.g., up to 400 ng depending on the kit implemented), which might affect the overall detection sensitivity of important ARGs (Petersen et al., 2019). Therefore, the potential of Nanopore SMS stool analysis should be evaluated in the near future with large and comparative studies, as we have done for the Illumina SMS in the present work.

## Data availability statement

The datasets presented in this study can be found in online repositories. The names of the repository/repositories and accession number(s) can be found at: https://www.ncbi.nlm.nih.gov/, PRJNA931234; https://www.ncbi.nlm.nih.gov/, PRJNA931814.

## Ethics statement

The studies involving humans were approved by Ethical Committee of the Canton Bern (ID: 2020-01683). The studies were conducted in accordance with the local legislation and institutional requirements. The participants provided their written informed consent to participate in this study.

## Author contributions

AE: conception and design. EC-M, CA, AM, PS, and AE: acquisition of data. EC-M and AE: drafting the work. All authors: analysis and interpretation of data, critical revision for important intellectual content, final approval of the version to be published, agreement to be accountable for all aspects of the work in ensuring that questions related to the accuracy or integrity of any part of the work are appropriately investigated and resolved.

## Funding

This work was supported by the Swiss National Science Foundation (SNF) grant No. 192514 (to AE). EC-M is a PhD student (2021–2024) supported by SNF.

## Conflict of interest

The authors declare that the research was conducted in the absence of any commercial or financial relationships that could be construed as a potential conflict of interest.

## Publisher’s note

All claims expressed in this article are solely those of the authors and do not necessarily represent those of their affiliated organizations, or those of the publisher, the editors and the reviewers. Any product that may be evaluated in this article, or claim that may be made by its manufacturer, is not guaranteed or endorsed by the publisher.

## Footnotes

^1^ https://data.snf.ch/grants/grant/192514

^2^ https://www.eucast.org/clinical_breakpoints

^3^ https://github.com/s-andrews/FastQC

^4^ https://github.com/usadellab/Trimmomatic

^5^ https://github.com/rrwick/Unicycler

^6^ https://sourceforge.net/projects/bbmap/

^7^ https://github.com/biobakery/kneaddata

^8^ https://github.com/lh3/seqtk

^9^ https://www.genomicepidemiology.org/

^10^ https://tygs.dsmz.de/

^11^ https://www.r-project.org/

^12^ https://github.com/arpcard/rgi

^13^ https://ccb.jhu.edu/software/kraken2/

^14^ https://ccb.jhu.edu/software/bracken/
